# A comparative analysis of student, educator, and simulated parent ratings of video-recorded medical student consultations in pediatrics

**DOI:** 10.1186/s41077-024-00282-7

**Published:** 2024-02-17

**Authors:** Clare C. Sullivan, Daire M. O’Leary, Fiona M. Boland, Claire M. Condron, Claire M. Mulhall, Walter J. Eppich

**Affiliations:** 1 RCSI SIM Centre for Simulation Education and Research, RCSI University of Medicine and Health Sciences, 123 St. Stephen’s Green, Dublin 2, Ireland; 2 Department of Pediatrics, RCSI University of Medicine and Health Sciences, 123 St. Stephen’s Green, Dublin 2, Ireland; 3 Data Science Centre, RCSI University of Medicine and Health Sciences, 123 St. Stephen’s Green, Dublin 2, Ireland; 4 Department of Medical Education & Collaborative Practice Centre, Faculty of Medicine, Dentistry and Health Sciences, The University of Melbourne, Melbourne, Australia

**Keywords:** Pediatrics, Simulation, Simulated participants, Communication skills, Feedback

## Abstract

**Background:**

Simulation-based education (SBE) affords learners opportunities to develop communication skills, including those related to pediatrics. Feedback is an integral part of SBE, and while much research into feedback from multiple sources exists, the findings are mixed. The aim of this comparative study was to replicate some of this work in a novel area, pediatric medical education, to better understand how multisource feedback (self, educator, and simulated parent) may inform learning and curriculum design.

**Methods:**

During their pediatric rotation, medical students participated in a consultation with a simulated parent, engaged in video-assisted self-reflection, and received feedback from both an educator and the simulated parent through an e-learning platform. The Pediatric Consultation Skills Assessment Tool (PCAT) was used for self-assessment and educator feedback, and the Consultation and Relational Empathy (CARE) measure was used for simulated parent feedback.

**Results:**

Our results showed that high-performing students underrated their performance, and low-performing students overrated their performance. Feedback from multiple sources helps to identify both areas of weakness in student performance and areas of weakness in student self-appraisal. Overall, general areas of weakness identified for the learners related to making contingency plans and providing easy-to-understand explanations for simulated parents. Some simulated parent feedback did not align with educator and student ratings, highlighting the value of including the simulated parent perspective. Our findings question whether a third party can reliably judge the simulated parent’s level of understanding.

**Conclusion:**

Multisource feedback allows students to develop layered insights into their performance and supports self-appraisal. Aggregating feedback through an e-learning platform allows educators to gain greater insights into the strengths and weakness of students and design a more tailored teaching plan to support student needs.

**Supplementary Information:**

The online version contains supplementary material available at 10.1186/s41077-024-00282-7.

## Background

Consultations with simulated participants (SPs), playing the role of patients or family members, allow students to practice communication skills, including those related to pediatrics, such as talking to parents, in a safe environment [1]. Much learning in simulation occurs post-simulation, making feedback or debriefing activities an integral part of simulation-based education (SBE) [2]. “Self-assessment as learning” is situated in theories of self-regulation and scaffolding of learning [3], and reflective observation is part of Kolb’s experiential learning theory [4]. While numerous studies have examined the alignment of learner self-assessment with feedback from other sources, such as faculty or SP, the existing evidence remains mixed. Many current studies on self-assessment have methodological flaws, specifically regarding the purpose and nature of the self-assessment [5]. Furthermore, methodological differences in the approach to comparisons, with some studies using paired comparisons and others using correlation, makes comparing the findings from different studies challenging [6].

Some studies identified learners’ moderate abilities to self-assess, with a trend to overestimate their ability on communication-based SP encounters [6]. Others also found a fair degree of agreement in scores but with learners underestimating their scores compared to faculty [7]. Further studies add to this discussion, reporting that only lower-performing students tend to overestimate their abilities, with high-performing students tending to underestimate their abilities [8], an effect referred to by some as the Dunning–Kruger effect [9]. This effect is under explored in medical consultations [10]. While age and gender across the same learner level do not appear to play a role in medical student’s ability to self-assess [11], learner level does appear to be an influencing factor, with learners improving as they progress through education [6].

SP perspectives on medical student communication skills can differ from educators’ [12]. In some cases, SPs tend to overestimate scores when compared to faculty [7], and learner self-assessment does not correlate with SP assessments [13].

Often, cognitive aids such as checklists, alongside a video of learner performance, are used to help scaffold self-assessment [3], and opportunities for informed self-assessment enable learners to integrate data from external sources to help them inform their self-assessment. However, these external sources must be from engaged and trustworthy sources for learners to value it in informing their self-assessment [14]. This perhaps explains why, despite the provision of external feedback, learner’s own opinions of themselves drive their learning goals [15].

The sole focus on the accuracy of self-assessment may be too narrow a view. Multisource feedback highlights differences in student and educator ratings, supports learning by prompting a gap analysis to inform areas for further development, and gives learners insight into their ability to self-appraise [16]. These gaps should be of interest to educators; if educators can better understand characteristics of learners who are poor at self-assessment or what areas students self-assess poorly, it can inform better design of educational activities. This aspect is especially relevant given the weight learners place on their own self-assessment in developing their future learning goals [15].

Influenced by these relative understandings of how multisource feedback informs student learning, we conducted a replication study of these effects in a relatively novel population. Studies concerning video-based self-assessment of communication skills in undergraduate pediatric medical education have involved real patients in clinical settings and focused on summative assessment. Students found the experience useful [17], they modified some of their behaviors [18], and their performance and ability to self-assess improved [19]. The challenges associated with teaching in the clinical setting, such as confidentiality and patient safety, can limit the scope of teaching involving real patients. Furthermore, summative assessment is often highly stressful for learners and has limited opportunities for feedback. To apply a different approach, we designed an SP-based intervention involving video-assisted self-assessment with both educator and simulated parent feedback through an e-learning platform. Developing a better understanding of the potential for multisource feedback to inform learning will allow us to enhance the design of curricula to optimize learning opportunities in SBE.

## Methods

This research took place at the RCSI University of Medicine and Health Sciences during the 2019–2020 academic year. The study was approved by the RCSI Research Ethics Committee REC001719.

### Intervention design

This study was part of a larger experiential learning intervention designed to support medical student learning of pediatric consultation skills [20]. Pediatric educators developed five pediatric consultation scenarios that included simulated parents (Table 1). In the intervention, students participated in a pediatric consultation with a simulated parent, self-assessed their performance, and received feedback from both an educator and a simulated parent. We focused on how feedback from multiple sources could support and encourage reflective practice to enhance both communication skills and insight into one’s own performance.

**Table 1:** Overview of scenarios

Simulated parent scenarios
Consultation with a parent of a 3-year-old child with asthma
Consultation with a parent of a 13-year-old child with diabetes
Consultation with a parent of 3-year-old child with transient synovitis
Consultation with a parent of a 2-year-old child with febrile seizures
Consultation with a parent of a 20-month-old child with isolated gross motor delay

### Subjects

All penultimate year medical students participated in this simulated consultation at the start of their pediatrics clinical rotation. Five different rotations of students began at different times throughout the year to account for the large learner numbers. Depending on the timing, students had already participated in 0–3 other clinical rotations (obstetrics and gynecology, general practice, medicine, and surgery or psychiatry). In previous years of their medical training, all students had participated in communication skills training involving SPs and were familiar with the format of teaching involving SPs. All students participated in the teaching intervention as a mandatory part of their curriculum, but only those who gave consent were included in the research data.

### Instruments

To provide students with a structured approach to reflection and multisource feedback for the simulated parent interactions, we used the published PCAT and CARE measure. The PCAT, designed to assess pediatricians’ communication skills in consultations [21], enabled self-assessment and educator feedback. A number of items were removed from the PCAT (Additional file 1: Appendix A) that were not applicable to the scenario, so that it aligned with the learning outcomes of the consultations (Additional file 2: Appendix B), leaving a total of 15 items used in this intervention. Each question had descriptive anchors specific to that item on a rating scale of 1 to 7. A higher score related to a better performance.

We selected the CARE measure for simulated parent feedback for two reasons: (a) the tool was designed specifically for patients to provide feedback about clinicians, and (b) the tool aligned well with the domains in which our simulated parents were trained to give feedback [22]. The CARE measure has 10 items which are rated on a 5-point scale from “poor” to “excellent,” where numerical scores of 1 to 5 are assigned to the ratings from poor to excellent. There is also a free text comment box at the end of the form.

### Faculty and SP training

Pediatric educators were familiarized with the modified PCAT to ensure a shared understanding of the tool and to agree upon expectations for student performance. All simulated parents involved had previously completed general RCSI SP training (Additional file 3: Appendix C). Before each simulation session, simulated parents had adequate time to review scenario scripts, discuss the CARE measure with faculty and the lead author (C. S.), and clarify any outstanding questions about the scenario scripts or the CARE measure. Ten simulated parents were trained in one of five different scenarios as outlined in Table 1.

### Educational intervention

In advance of the teaching day, patient referral letters with links to relevant preparatory reading materials were available to students on an e-learning platform (Moodle, West Perth, Australia). On the teaching day, pediatric educators explained the learning objectives and clarified students’ questions during the pre-briefs. Students participated individually in one 8-min consultation involving a simulated parent for which the child was not present. After the scenario ended, the simulated parent had 2 min to give the student verbal feedback in the consultation room. For the verbal feedback, the simulated parents were trained to invite the student perspective, give concise feedback from their own perspective based on communication skills, and invite one or two peers who were observing in the room to contribute feedback also. Each student encountered a different scenario with a different simulated parent.

### Data collection

**Fig. 1:**
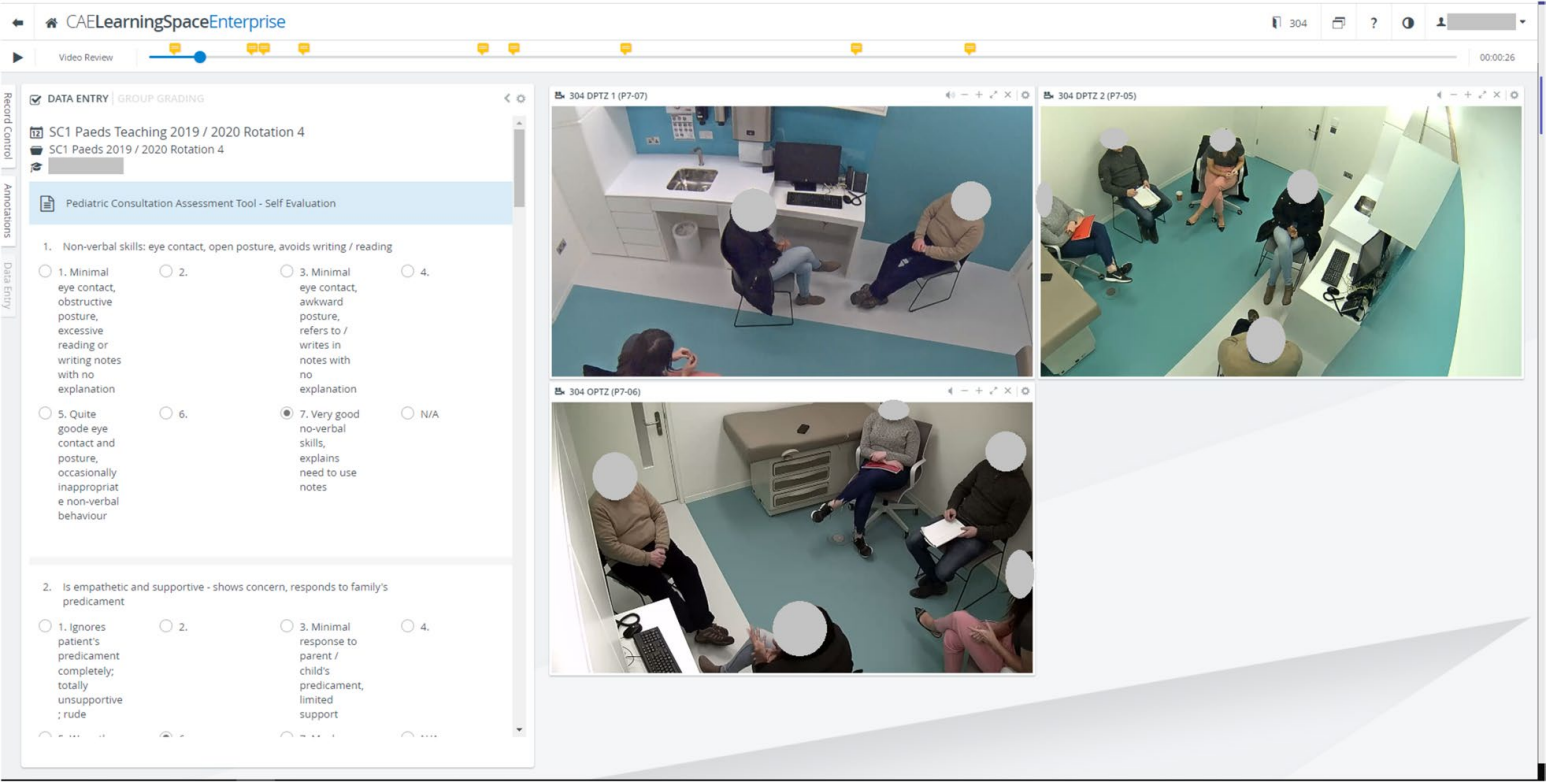
A screenshot from the CAE LearningSpace software shows recording and checklist on the left

Immediately after leaving the consultation room, the simulated parents completed the CARE measure. Following the consultations, the lead researcher (C. S.) showed the students how to access their videos and complete the PCAT on the e-learning platform (CAE LearningSpace, Sarsota, FL, USA). The students were given 30 min of dedicated time to watch their video while completing questions from the PCAT (Fig. 1). Faculty viewed and rated video recordings of the learning scenarios using the PCAT through the e-learning platform on the same day. The students were able to view both educator and simulated parent ratings after they had submitted their own ratings. Further, students could discuss any questions or concerns with educators later during the teaching day.

Thirty percent of learner videos were marked by a second rater (CS) to calculate the inter-rater reliability for the PCAT [23]. Missing data were given the lowest value on the scale for both measures because these occurred most often when a student had missed that element of the consultation.

### Statistical analysis

Data were exported from the e-learning platform for analysis using the R statistical software (R Core Team, 2019, Vienna). The internal consistency of the scales was measured using Cronbach’s alpha [24]. Inter-rater reliability for the PCAT was calculated using the quadratic weighted kappa score [23].

#### PCAT

Educator and student ratings on the PCAT were compared in a number of different ways [6]: (a) Spearman correlation coefficient was used to measure the correlation between educator and student rated scores; (b) paired *t*-tests were used to compare the scores of the two groups at the statistical significance level *p* < 0.05, and this was completed on the whole group and also on assigned quartiles according to their educator rating, similar to the method used by Mort, Hansen [8]; (c) the bias towards overrating/underrating was assessed by looking at the total percentage of students who rated above and below educator, by whole group, and by assigned quartile.

For the itemized analysis, the median and interquartile range were calculated for each item to identify areas of strength or weakness in the students’ performance. To identify the gap between educator and student ratings, we identified for each item the total number of students who rated themselves within one mark of the educator, similar to a method used previously [16]. Our aim was twofold: (i) to identify areas of student weakness, items with a low median rating, and (ii) to identify areas of weakness in student self-appraisal, items with poor agreement between student and educator rating.

#### CARE

For the CARE measure, we used the Spearman correlation coefficient to assess the correlation of the overall score on the CARE measure with the overall student rating and educator rating on the PCAT. We also looked at the itemized scores on the CARE measure to identify areas of weakness as identified by the simulated parents. 

## Results

Fifty-one percent (144/278) of penultimate year medical students consented to participate in the research, and 127/144 (88%) completed the PCAT self-assessment. We repeated the intervention across four different groups. Thirty-one percent (40) of students participated in September 2019, 28 (22%) students participated in November 2019, 43 (34%) students participated in January 2020, and 16 (13%) students participated in March 2020. The fifth rotation was excluded from analysis due to significant changes in the teaching resulting from the COVID-19 pandemic. Six educators rated between 9 and 26 videos.

In terms of inter-rater reliability, the quadratic weighted kappa score for the PCAT was 0.641 (40 videos × 15 PCAT items = 600), which indicates substantial agreement between raters [23]. The value for Cronbach’s alpha for the PCAT was 0.87 and 0.86 for the educator and student ratings respectively and 0.90 for the CARE measure, indicating good internal consistency across both scales [24].

### Comparison of educator and student rating PCAT

The student ratings were compared to the educator ratings for the total rating (sum of the items) for the group as a whole and by quartile (*n* = 127) (Table 2). The mean total educator-assessed student score was 76.8, and the mean total student self-assessed score was 76.4. There was no statistically significant difference between educator and student ratings for the total score (*p* = 0.654 *t*-test) (Table 2). Slightly, more students rated themselves higher than the educator rating on the total score (50%) than rated themselves lower (47%), and 3% of students gave themselves the same total score as the educator. There was a small positive correlation of 0.3 (Pearson) between the student and educator scores. The weakest students (first quartile) had the highest percentage of students (78%) rating themselves higher than the educator rating, and this difference reached statistical significance (*p* < 0.001). The strongest students (fourth quartile) had the highest percentage of students rating themselves lower than educator (74%), and this difference also reached statistical significance (*p* < 0.001) (Table 2).

**Table 2:** PCAT aggregate student and educator ratings

Group	Number of students	Student rating mean (sd)	Educator rating Mean (sd)	*p*-value (paired *t*-test)	Student ratings compared to educator ratings			Correlation coefficient (Pearson)	*p*-value (significance of correlation)
					**% above**	**% equal**	**% below**		
Overall	127	76.4 (10.6)	76.8 (9)	0.654	64 (50%)	4 (3%)	59 (46%)	0.30	< 0.001
Q1 < 25	32	73.1 (9.7)	65.4 (4.9)	< 0.001*	25 (78%)	2 (6%)	5 (16%)	0.18	0.324
Q2 25 − 50	32	72.8 (12.5)	74.5 (1.9)	0.425	16 (50%)	1 (3%)	15 (47%)	0.30	0.095
Q3 > 50 − 75	32	78.9 (8.7)	79.6 (1.6)	0.654	15 (47%)	1 (3%)	16 (50%)	0.15	0.413
Q4 > 75	31	80.8 (9.2)	88.2 (4.5)	< 0.001*	8 (26%)	0 (0%)	23 (74%)	− 0.14	0.452
Incomplete	17	N/A	74.8 (8.8)	N/A	N/A	N/A	N/A	N/A	N/A

Aggregate rating: sum of the items (15 items on PCAT)

Mean and standard deviation (sd) used for aggregate scores

Q1, Q2, Q3, and Q4, student quartiles based on educator ratings

Paired *t*-test — comparison of means

^*^ Significant at the *p* < 0.05 level

Correlation measure, Pearson correlation coefficient

Significance of correlation, test if correlation significantly different from 0

Incomplete, observations where student did not complete their own rating

### Itemized analysis PCAT

**Fig. 2:**
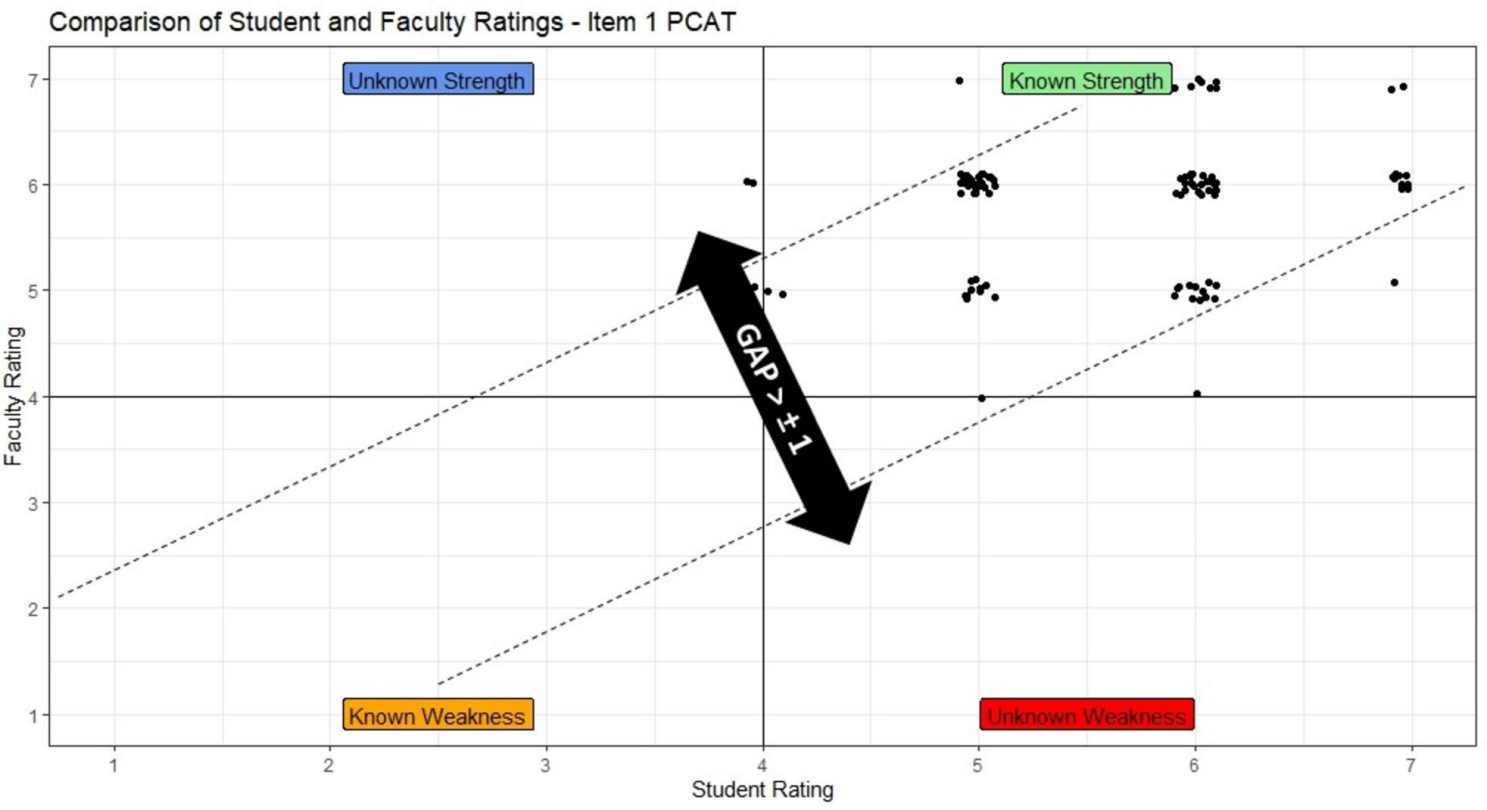
Scatterplot of educator and student ratings item1 PCAT — good alignment between educator and student ratings

**Fig. 3:**
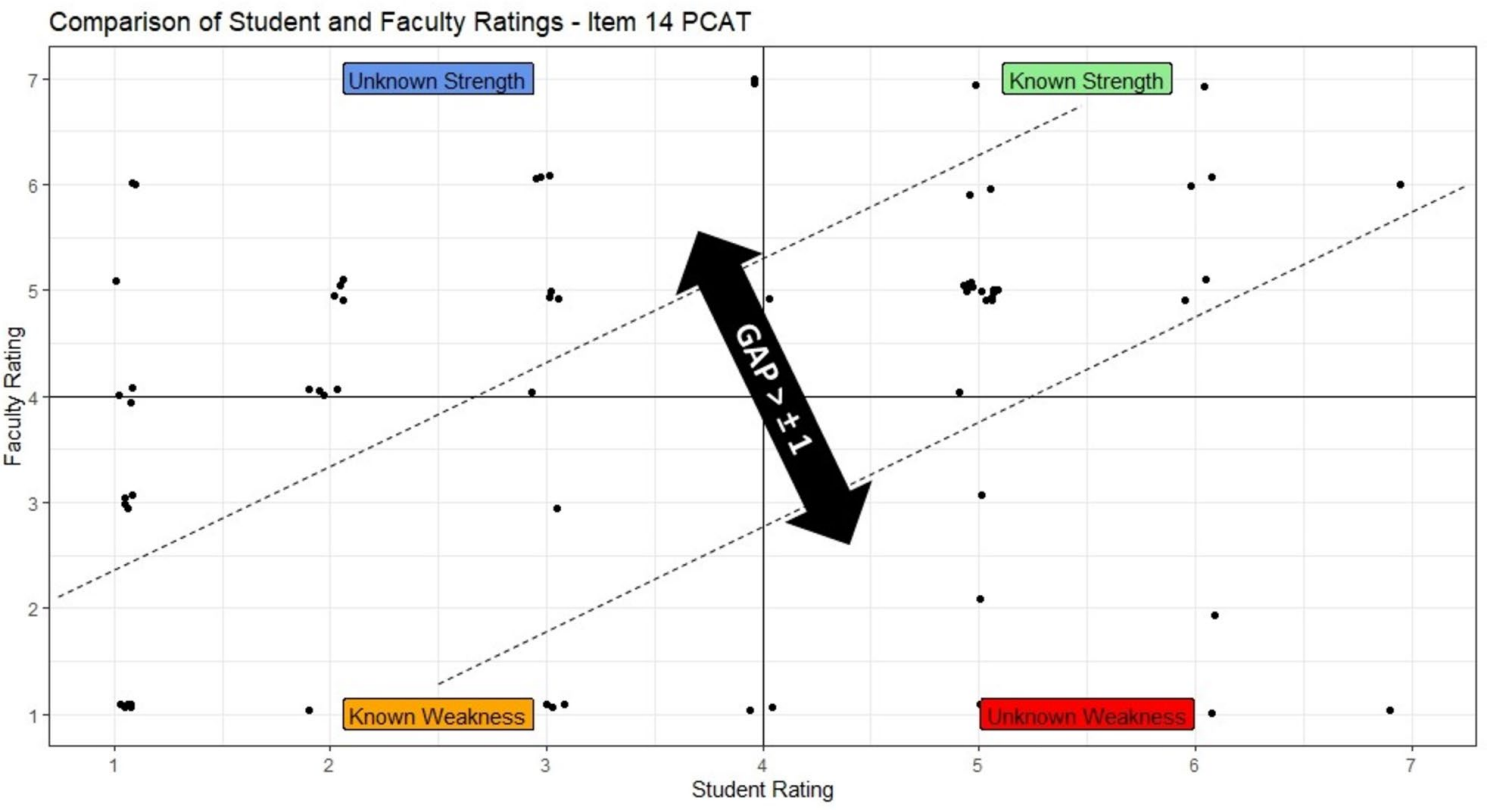
Scatterplot of educator and student ratings  item 14 — poor alignment between educator and student ratings

We calculated the percentage of students rating themselves within one mark of the educator rating for each of the 15 items, to identify the degree of accuracy in student self-assessment (Figs. 2 and 3). Sixty-five percent (83) of students rated 11 or more questions, 32% (40) of students rated between 6 and 10 questions, and 3% (4) of students rated 5 or fewer questions within one mark of the educator rating.

**Fig. 4:**
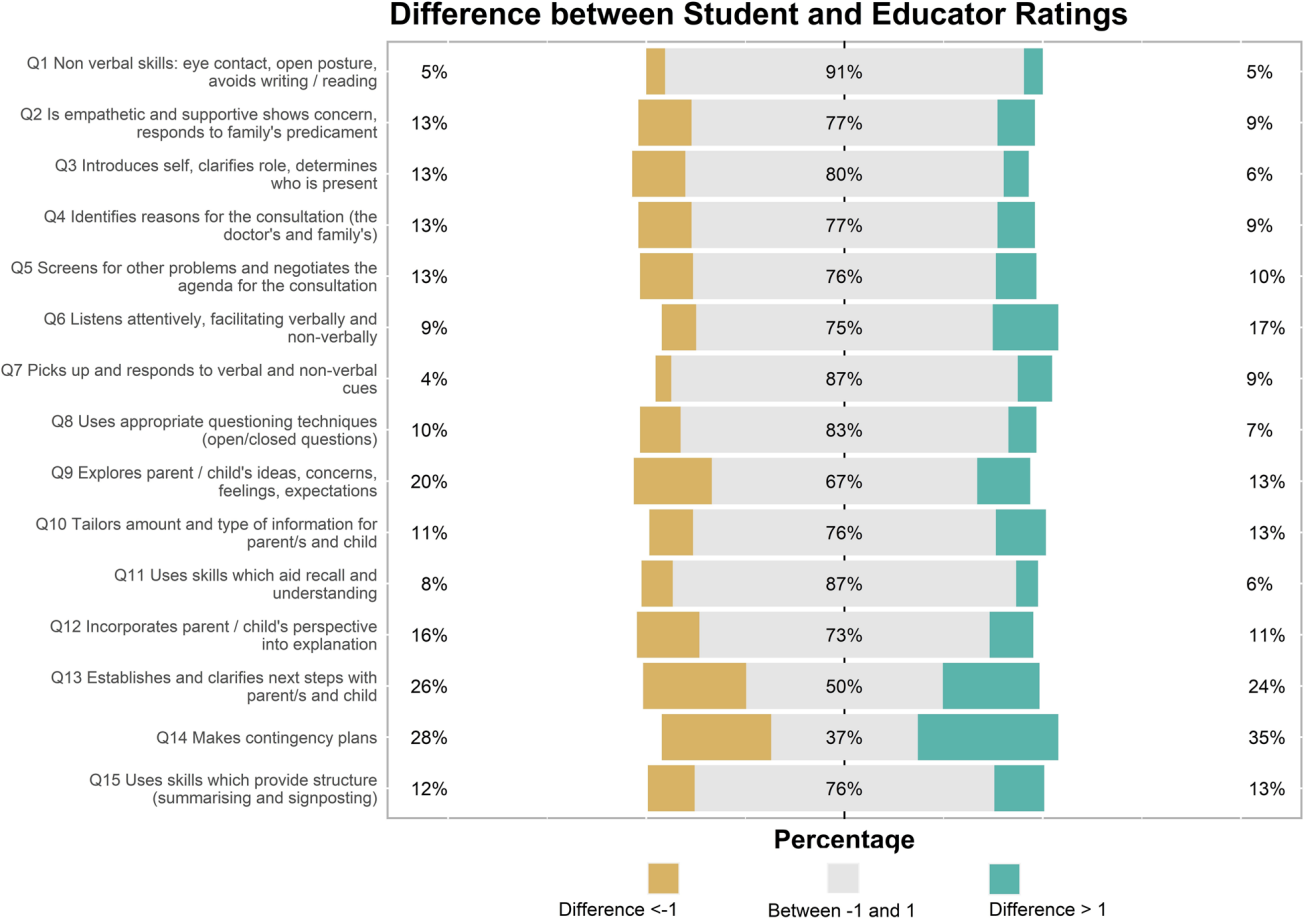
Comparison between student and educator rated items on the PCAT

Over 85% of students rated themselves within one mark of the educator rating for item 1 (*nonverbal skills*), item 7 (*responding to cues*), and item 11 (*recall and understanding*). The questions with the fewest student ratings within one mark of the educator rating were item 14 (37%) (*makes contingency plans*), item 13 (50%) (*establishes and clarifies next steps*), and item 9 (67%) (*explores parents’ ideas, concerns, expectations*). All other items had greater than 70% of student ratings within one mark of the educator rating (Table 3 and Fig. 4).

**Table 3:** PCAT itemized result for student and educator ratings

Item	Student rating median (IQR)	Educator rating median (IQR)	% + / − 1 from educator	Item details
1	6 (5 to 6)	6 (5 to 6)	91%	Nonverbal skills: eye contact, open posture, avoids writing/reading
2	6 (5 to 6)	6 (5 to 6)	77%	Is empathetic and supportive, shows concern, responds to family’s predicament
3	6 (5 to 7)	6 (5 to 6)	80%	Introduces self, clarifies role, determines who is present
4	6 (5 to 6)	6 (5 to 6)	77%	Identifies reasons for the consultation (the doctor’s and family’s)
5	5 (4.5 to 6)	5 (5 to 6)	76%	Screens for other problems and negotiates the agenda for the consultation
6	6 (5 to 7)	6 (5 to 6)	75%	Listens attentively, facilitating verbally and nonverbally
7	5 (5 to 6)	5 (5 to 6)	87%	Picks up and responds to verbal and nonverbal cues
8	5 (5 to 6)	5 (5 to 6)	83%	Uses appropriate questioning techniques (open/closed questions)
9	5 (4.5 to 6)	5 (5 to 6)	67%	Explores parent/child’s ideas, concerns, feelings, expectations
10	5 (5 to 5)	5 (5 to 6)	76%	Tailors amount and type of information for parent/s and child
11	5 (5 to 6)	5 (5 to 6)	87%	Uses skills which aid recall and understanding
12	5 (4 to 5)	5 (5 to 6)	73%	Incorporates parent/child’s perspective into explanation
13	5 (3 to 5.5)	5 (3 to 5)	50%	Establishes and clarifies next steps with parent/s and child
14	4 (1 to 5)	2 (1 to 5)	37%	Makes contingency plans
15	5 (4 to 5)	5 (4 to 6)	76%	Uses skills which provide structure (summarizing and signposting)

*n* = 127

Scale: 1–7, with 1 representing the poorest performance and 7 representing the best performance. Descriptors relevant to each question present for ratings 1, 3, 5, and 7

Mean and inter-quartile range (IQR) used to describe responses as scale was ordinal

*GAP*, difference of + / − 1 from educator rating

### Simulated parent rating CARE

The mean (standard deviation) total score for learner performance as rated by the simulated parents using the CARE measure was 31.4 (7.5) out of 50 (Table 4). Comparing the simulated parent ratings to the student quartiles, the simulated parents appeared to rate the high-performing students higher and the low-performing students lower (Table 4). There was a moderate positive correlation between simulated parent rating using the CARE measure and educator rating using the PCAT (0.47) which was higher than the correlation between simulated parent rating on the CARE measure and student self-rating on the PCAT (0.33).

**Table 4:** Comparison of aggregate ratings for CARE measure and PCAT

**Group**	**Number of students**	**SP rating CARE** **Mean (SD)**	**Educator rating PCAT** **Mean (SD)**	**Correlation SP — educator rating**	***p*-value** **(sig. of correlation: SP — educator)**	**Student rating mean (SD)**	**Correlation SP — student rating**	***p*-value** **(sig. of correlation SP — student)**
Overall	127	31.4 (7.5)	76.8 (9)	0.47	< 0.001	76.4 (10.6)	0.33	< 0.001
Q1 < 25	32	27.0 (7.2)	65.4 (4.9)	0.50	0.004	73.1 (9.7)	0.31	0.084
Q2 25–50	32	30.0 (6.8)	74.5 (1.9)	0.05	0.786	72.8 (12.5)	0.37	0.037
Q3 > 50–75	32	33.4 (7.3)	79.6 (1.6)	0.01	0.957	78.9 (8.7)	0.39	0.027
Q4 > 75	31	35.3 (6.2)	88.2 (4.5)	0.23	0.915	80.8 (9.2)	–0.21	0.257
Incomplete	17	26.3 (8.6)	74.8 (8.8)	0.23	0.374	N/A	N/A	N/A

Aggregate rating: sum of the items (10 items on CARE measure) (15 items on PCAT)

Mean and standard deviation (sd) used for aggregate scores

Correlation measure, Pearson correlation coefficient

Incomplete, students who did not complete their own review

Simulated parents rated students highest on three items (median 4, *IQR* 3 to 4, 5-point scale): item 1, *making them feel at ease*; item 2, *letting them tell their story*; and item 3, *really listening*. Simulated parents rated students lowest on item 9, *helping you take control* (median 1, *IQR* 1 to 3); on item 8, *explaining things clearly* (median 2, *IQR* 2 to 3); and item 10, *making a plan of action* (median 2, *IQR* 1 to 3) (Table 5).

**Table 5:** Itemized SP ratings on the CARE measure

Item	Median (IQR)	Item details
1	4 (3 to 4)	Q1: Making you feel at ease
2	4 (3 to 4)	Q2: Letting you tell your story
3	4 (3 to 4)	Q3: Really listening
4	3 (2 to 4)	Q4: Being interested in you
5	3 (3 to 4)	Q5: Fully understanding your concerns
6	3 (3 to 4)	Q6: Showing care and compassion
7	3 (3 to 4)	Q7: Being positive
8	2 (2 to 3)	Q8: Explaining things clearly
9	1 (1 to 3)	Q9: Helping you to take control
10	2 (1 to 3)	Q10: Making a plan of action with you

*n* = 127

Scale: 5-point scale with 1 = poor to 5 = excellent

Mean and inter-quartile range (IQR) used to describe responses as scale was ordinal

## Discussion

To our knowledge, our study is the first in undergraduate pediatric medical education to (a) assess if patterns of feedback from multiple sources reflect those studied in other areas of medical education and (b) examine how these sources of feedback may contribute to learning in a pediatric context. By replicating similar studies in other domains of medical education, our study adds to the ongoing debate about the benefits and challenges associated with self-assessment. In terms of the direction of learner self-assessment, some studies found that medical students overestimate their performance compared with educator ratings [6] and others found learners underestimate [7]. Our study fully replicates neither as we had approximately equal numbers of students who overestimated as underestimated their performance. However, as we delved further into our results, we found evidence in our data that stronger students underrated their performance and weaker students overrated their performance, a pattern in line with what some refer to as the Dunning-Kruger effect [9]. Research into qualified physician self-appraisal has found poor self-assessment accuracy also showing this effect, those whose performance was in the lowest quartile overestimated their ability, and those in the highest quartile underestimated their ability [25]. While little research has examined this effect in undergraduate medical education (UME), the concept has been identified in undergraduate pharmacy education [8].

Educators should consider this important effect when designing SBE teaching involving self-assessment. As Calhoun et al. (2009) describe, by assessing student ability in both communication skills and self-appraisal, educators can identify areas in most urgent need of support, namely those areas in which students have poor performance but high self-appraisal, as these areas represent a blind spot of unrecognized weakness, which is unlikely to change without intervention. Informed self-assessment [14], whereby a learner’s self-assessment is supported by feedback from other sources, can be extremely valuable for learners. This process helps learners develop insight into their weaknesses and helps broaden feedback when multiple sources of feedback are provided, reducing potential bias from any one source. It can also help learners to develop the ability to identify their own mistakes and self-regulate their learning [26].

The itemized comparison of ratings for the PCAT showed areas in which students performed poorly and areas in which self-assessment differed from educators. According to educator ratings, students performed well on all items (median 5 or above) except for item 14 (median 2), *making a contingency plan*. The student self-assessments showed a similar pattern. The item related to *making a contingency plan* also had the poorest level of agreement between educator and student ratings (34% of students within one mark of educator) and the largest variation in both educator and student rated scores. While these findings perhaps indicate some confusion around this question’s interpretation, they also suggest a need for further support and training in this area since it highlights possible differences in learner and SP perspectives of what constitutes a plan.

Educator feedback is the norm in simulation and widely acknowledged to be essential in medical education. A growing evidence base also values the importance of patient and caregiver perspectives as sources of student feedback to foster a holistic approach to communication skills training. For example, poor attention to further planning during the consultation was supported by the simulated parent feedback. The CARE measure item, related to *making a plan of action with you*, was the second lowest rated item by the simulated parents. “Making a plan” was a new skill for students in their penultimate year, one reason why some students may not have approached it well.

Some simulated parent feedback did not align with educator and student ratings. The simulated parent ratings identified poor performance on question 8, *explaining things clearly*, and question 9, *helping you take control*, skills that require the student to consider the other person’s perspective. However, educator and student ratings relating to explaining and understanding on the PCAT indicated a good performance, suggesting that they lacked insight into how clear the simulated parents found student explanations. This finding highlights the value of including the simulated parent perspective in feedback and demonstrates how students can miss valuable feedback unless the simulated parent perspective is integrated into the teaching activity. It brings into question whether a third party can reliably judge the degree to which a patient has understood explanations. This finding also questions clinicians’ level of understanding of their patient’s needs, given the lack of correlation in learner and SP scores from studies with residents [13]. Additional training for students to explore the patient’s perspective may be beneficial to our students.

Our findings have important implications for the design of SBE activities. Optimal SBE learning experiences go beyond simulation scenarios alone but are supported by other activities including demonstrations, feedback, and peer observation which help learners gain competence, insight, and confidence in their own ability. Our findings also highlight how looking at correlation and alignment alone does not show the true value of feedback from multiple sources. Rather than the alignment, we believe the missalignment shines a light on real learning opportunities; the challenge lies in helping learners value these differences and integrate these insights into future learning goals.

Our findings advance our understanding of the Dunning-Kruger effect in UME and help explain the vital role of these activities which support the simulation activity itself. Weak students overrate their performance due to limited knowledge which also results in a lack of insight since knowledge forms the basis for insight. Weaker students who are overconfident may fail to adequately prepare and underperform. Stronger students may have a poor experience since confidence fosters more positive sentiments towards an activity [9]. Based on our findings, these two groups may require different approaches to feedback. While strong performers can derive insight and confidence from observing their peers, weaker students may fail to gain similar benefit due to their diminished ability to identify whether a performance is better or worse than their own [10]. Weaker students are more likely to react unfavorably to feedback and need education and feedback from multiple sources to gain perspective on their performance [9]. Therefore, well-designed SBE activities provide learning opportunities which meet the needs of both groups.

We sought to identify how feedback from multiple sources (self, educator, and simulated parent), in the context of undergraduate pediatric consultation skills, can inform learning and curriculum design. In doing so, we add to the wider understanding of the relationships between different sources of feedback. Our findings demonstrate how multisource feedback in SBE contributes to a broader learning experience, allowing learners to easily identify where their self-appraisal differs from that of other raters. Further, gaps in global student ability can inform iterative curricula design. Simulated parent feedback can help students understand the skills they need to remain patient-centered. By recording simulated consultations and giving learners access to their video and feedback, students may review their performance again and continue to reflect after the simulation itself. This approach would also allow educators to provide more in-depth feedback to promote learning and improvement outside the physical space of the simulation center. Future research will clarify the impact of later review of performance in simulation.

### Limitations

Our study has several limitations. We did not collect demographic data on participants, so we could not perform analysis to identify differences based on gender or age nor could we describe our population in detail. We removed items not relevant to the scenario from the PCAT which may have affected the psychometric properties of the tool; it was not within the scope of the study to revalidate the measure. While all students participated in the educational intervention, only student data for those who consented were included in the study; therefore, the results are only representative of those who participated and may not be generalizable to other groups. The large sample size however should reduce bias and diminish the impact of outliers. Learners received brief verbal feedback from the SPs prior to completing their self-assessment. The SP comments did not appear to align with the learner ratings, indicating limited influence of this verbal feedback. We specified a difference of + / − 1 as a gap in the student ability to self-assess as described by Calhoun et al. (2009) [16]. However, they identify themselves that this cutoff requires further analysis to confirm that it is an optimal. Further rater training could have achieved a higher inter-rater reliability. We used two separate tools to review student performance, the PCAT (faculty and learners) and the CARE measure (SPs). These have not previously been compared so the baseline correlation between these tools is unknown. Some of the constructs described in these tools were similar, but others differed therefore influencing their correlation.

## Conclusion

Multisource feedback in UME has the potential to enhance learning in simulated consultations. We have shown how feedback from multiple sources can differ, and these different perspectives can promote learning. Students in this study had the greatest difficulty making contingency plans and providing parents with easy-to-understand explanations. The latter weakness is one simulated parents appear best positioned to identify. Comparing different sources of feedback allows students to develop insight into their own ability to self-appraise, an increasingly important skill for them as they progress in their training and onto professional practice. Multisource feedback allows educators gain greater insights into the strengths and weaknesses of students and make a more tailored teaching plan.

### Supplementary Information

**Additional file 1: Appendix A.** Subset of Questions from the Paediatric Consultation Skills Assessment Tool (PCAT) (Howells et al. 2010).

**Additional file 2: Appendix B.** Learning Objectives.

**Additional file 3: Appendix C.** Simulated Participant (SP) Training Overview.

## Data Availability

The datasets used and/or analyzed during the current study are available from the corresponding author on reasonable request.
