# Supplementary material for: A comparative analysis of student, educator, and simulated parent ratings of video-recorded medical student consultations in pediatrics
Source: Adv Simul (Lond). 2024 Feb 17;9:10. doi: 10.1186/s41077-024-00282-7 (PMC10874056; doi:10.1186/s41077-024-00282-7)
Supplement: Supplementary file 2 — Additional file 2: Appendix B. Learning Objectives. [file 41077_2024_282_MOESM2_ESM.docx]

**Appendix B**

**Learning Objectives**

| **Course Element** | **Learning Outcomes** |
| --- | --- |
| Recorded simulated parent consultations (without a child present) with multi-source feedback (self, faculty and simulated parent) | Correctly demonstrates specific tasks of an effective consultation.   1. Establishes and builds a relationship. 2. Initiates the consultation and sets the agenda. 3. Establishes, recognises, and meets patient needs. 4. Gathers information. 5. Explains the diagnosis and plans and negotiates management plans. 6. Structures, and prioritizes the consultation. 7. Closes the consultation and establishes future plan. |
