# Supplementary material for: A comparative analysis of student, educator, and simulated parent ratings of video-recorded medical student consultations in pediatrics
Source: Adv Simul (Lond). 2024 Feb 17;9:10. doi: 10.1186/s41077-024-00282-7 (PMC10874056; doi:10.1186/s41077-024-00282-7)
Supplement: Supplementary file 3 — Additional file 3: Appendix C. Simulated Participant (SP) Training Overview. [file 41077_2024_282_MOESM3_ESM.docx]

**Appendix C**

**Simulated Participant (SP) Training Overview**

| **RCSI Simulated Participant Training Programme** | |
| --- | --- |
| Session 1 | Professionalism  Concept of simulation  Simulation in healthcare education  Introduction to cases |
| Session 2 | Healthcare consultations and curriculum overview  Attributes of a good SP  SP case development – the backstory  Communication skills |
| Session 3 | Hybrid simulation  Feedback  Confidentiality  Calgary Cambridge model  Case practice |
| Session 4 | SP performance assessment  How to portray emotions  Review |
